# The Potential Cost and Cost-Effectiveness Impact of Using a Machine Learning Algorithm for Early Detection of Sepsis in Intensive Care Units in Sweden

**DOI:** 10.36469/jheor.2022.33951

**Published:** 2022-04-26

**Authors:** Oskar Ericson, Jonas Hjelmgren, Fredrik Sjövall, Joakim Söderberg, Inger Persson

**Affiliations:** 1 The Swedish Institute for Health Economics (IHE), Lund, Sweden; 2 Department of Intensive and Perioperative Medicine Skåne University Hospital, Malmö, Sweden; 3 AlgoDx AB, Stockholm, Sweden; 4 Department of Statistics Uppsala University

**Keywords:** sepsis, machine learning, early detection, costs, economic modeling, intensive care

## Abstract

**Background:** Early diagnosis of sepsis has been shown to reduce treatment delays, increase appropriate care, and reduce mortality. The sepsis machine learning algorithm NAVOY® Sepsis, based on variables routinely collected at intensive care units (ICUs), has shown excellent predictive properties. However, the economic consequences of forecasting the onset of sepsis are unknown.

**Objectives:** The potential cost and cost-effectiveness impact of a machine learning algorithm forecasting the onset of sepsis was estimated in an ICU setting.

**Methods:** A health economic model has been developed to capture short-term and long-term consequences of sepsis. The model is based on findings from a randomized, prospective clinical evaluation of NAVOY® Sepsis and from literature sources. Modeling the relationship between time from sepsis onset to treatment and prevalence of septic shock and in-hospital mortality were of particular interest. The model base case assumes that the time to treatment coincides with the time to detection and that the algorithm predicts sepsis 3 hours prior to onset. Total costs include the costs of the prediction algorithm, days spent at the ICU and hospital ward, and long-term consequences. Costs are estimated for an average patient admitted to the ICU and for the healthcare system. The reference method is sepsis diagnosis in accordance with clinical practice.

**Results:** In Sweden, the total cost per patient amounts to €16 436 and €16 512 for the algorithm and current practice arms, respectively, implying a potential cost saving per patient of €76. The largest cost saving is for the ICU stay, which is reduced by 0.16 days per patient (5860 ICU days for the healthcare sector) resulting in a cost saving of €1009 per ICU patient. Stochastic scenario analysis showed that NAVOY® Sepsis was a dominant treatment option in most scenarios and well below an established threshold of €20 000 per quality-adjusted life-year. A 3-hour faster detection implies a reduction in in-hospital mortality, resulting in 356 lives saved per year.

**Conclusions:** A sepsis prediction algorithm such as NAVOY® Sepsis reduces the cost per ICU patient and will potentially have a substantial cost-saving and life-saving impact for ICU departments and the healthcare system.

## INTRODUCTION

Sepsis is a potentially life-threatening condition in which initial signs of disease can be difficult for healthcare professionals to interpret. Approximately 6% of patients admitted to hospital emergency departments and around 30% of patients that are admitted to ICU wards have signs of sepsis.^1,2^ Sepsis arises when the host response to infection causes organ dysfunction in the patient and is persistently associated with a high mortality of 20%-30%. Globally, sepsis affects about 30 million people every year.^3^ The condition of some of these patients will deteriorate to septic shock (ie, the inability to sustain normal blood pressure), which is associated with an even higher mortality rate of 30%-40%.^2^ Also, patients admitted to the ICU for reasons other than sepsis are at high risk of developing sepsis during their ICU stay. For example, during the COVID-19 pandemic, it was established in Wuhan that sepsis was the most frequently observed complication among adult inpatients who died during hospitalization with confirmed COVID-19.^4^

Timing is critical when treating sepsis. Early diagnosis of sepsis has been shown to reduce delays in treatment, increase appropriate care, and reduce mortality.^5–8^ Early intervention with fluids, antibiotics, source control, and organ-supportive measures will improve patient outcomes.

A recently published review of 37 cost-of-illness studies published between 2005 and 2015 found that the mean hospital cost per patient stay for sepsis ranged between $13 292 (€10 909) and $75 015 (€61 567), using the January 22, 2021, exchange rate of €/$ = 0.82).^9^ The medians of the mean reported hospital costs indicated higher cost for survivors ($34 855 [€28 607]) compared with nonsurvivors ($20 537 [€16 855]). Also, the hospital cost for severe sepsis and septic shock was higher ($46 834 [€38 438]), compared with septicemia ($20 740 [€17 022]). Previous studies have shown that the timing of sepsis treatment has an impact on sepsis mortality. However, the cost impact of early detection and treatment of sepsis is less known.^10^

Despite updated diagnostic criteria, early detection of sepsis is still complicated by the lack of reliable biomarkers.^11^ A large number of biomarkers have been evaluated clinically as possible prognostic markers for sepsis; procalcitonin and C-reactive protein have been most frequently investigated.^12^ Other biomarkers include molecules related to vascular endothelial damage, coagulation, and vasodilation; cytokine/chemokines; heparin-binding proteins; acute-phase proteins; and many others.^12–14^ None of these, alone or in combination, have demonstrated high enough sensitivity or specificity to be routinely used in clinical practice.^12^ In this context, there exists significant room for improvement for alternative diagnostic approaches.

In practice, sepsis remains a clinical diagnosis made by combining information from physical examinations with laboratory data and information from monitoring devices. This procedure is time consuming, subjective, and heavily dependent on the skills and experience of the physician. These manual routines therefore increase the risk of a prolonged time to diagnosis.

Much of the same data, and more, that are now processed to make a sepsis diagnosis can be continuously interpreted by a machine learning prediction algorithm. This type of algorithm has a large potential to improve early detection of sepsis and thereby improve patient outcomes.

AlgoDx AB (Stockholm, Sweden) has developed NAVOY® Sepsis,^15^ a sepsis prediction algorithm with excellent predictive properties, based on variables routinely collected at ICUs. This algorithm has been validated in a prospective randomized clinical trial (ClinicalTrials.gov identifier: NCT04570618; manuscript in preparation) and is CE-marked as Software as a Medical Device, designed for commercial use in European ICUs.

In this study, we investigated the cost-saving potential of a machine learning algorithm such as NAVOY® Sepsis for early detection of sepsis in intensive care units (ICUs) in Sweden. Since the evaluation of costs and cost-effectiveness of novel diagnostic tools depend on multiple factors such as the definition of sepsis, the diagnostic tool currently used, and the sensitivity and specificity of the diagnostic tool,^16^ we aimed at developing a health economic model that would enable us to simulate the cost-saving potential of a sepsis prediction algorithm in various contexts.

## METHODS

### Model Structure

**Figure 1.**
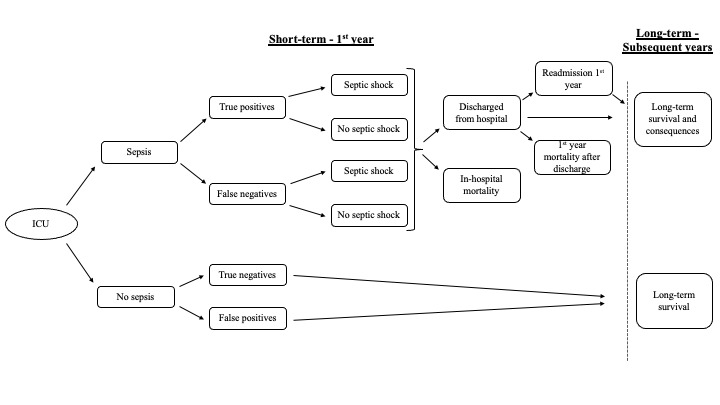
Model Structure for a Patient Population Not Diagnosed With Sepsis at Admission Abbreviation: ICU, intensive care unit.

A health economic model with a decision tree structure (Figure 1) has been developed to capture short-term (≤1 year) and long-term (>1 year) effects of sepsis. Short-term parameters for the model included incidence of suspected sepsis in the ICU, sensitivity and specificity of the predictive method (algorithm) or diagnostic method (current practice), the probability of septic shock and in-hospital mortality (affected by time to sepsis detection and time to adequate antibiotic therapy), readmission rate after discharge, and first-year mortality after discharge. The direct effect of introducing a new prediction algorithm was modeled by the time to sepsis detection, keeping the time to adequate antibiotic therapy the same across different predictive or diagnostic algorithms. Long-term parameters for subsequent years included long-term survival and long-term consequences.

### Data

The model was informed by the available literature. Base case inputs are summarized in Table 1. The relevant patient group for this assessment was all adult patients who were not diagnosed with sepsis at the time of admission. At the beginning of the model, sepsis incidence in ICUs was identified by distributing patients to either the “sepsis” or “no sepsis” health state (Figure 1). In the model, the incidence of sepsis in ICUs was defined as the proportion of patients without sepsis who develop sepsis after admission, estimated to be 14.1% in the base case in accordance with the findings of Sakr et al.^2^ Patients admitted with sepsis were therefore not included in the model. These patients can develop a secondary sepsis infection during the ICU stay,^17^ and a prediction algorithm would certainly apply to this group of patients. For the purpose of the analysis, however, we focused only on the patients without sepsis at diagnosis.

**Table 1.** Model Base Case Inputs

**Input Variable**	**Sepsis Prediction Algorithm**	**Clinical Practice**
Age	60 years (Karlsson et al^18^)	
Incidence	14.1% (Sakr et al^2^)	
Sensitivity/specificity	80.0%/85.1% (NAVOY® Sepsis)	79.2%/78.5% (Lengquist et al^19^)
Total time to sepsis treatment (compared with time of diagnosis for SOC)	True positives -3 hours	True positives 0 hours
	False negatives +3 hours	False negatives +3 hours
Proportion septic shock when detection is set 3 hours earlier than SOC	40% (assumption)	
Proportion septic shock SOC (at 0 hours)	56.6% (Sakr et al^2^)	
In-hospital mortality	True positives (-3 hours) Septic shock, 39.5% No septic shock, 0% False negatives (+3 hours) Septic shock, 46.4% No septic shock, 1.6% (Ferrer et al^10^)	True positives (0 hours) Septic shock, 42.8% No septic shock, 0% False negatives (+3 hours) Septic shock, 46.4% No septic shock, 1.6% (Ferrer et al^10^)
Postdischarge mortality first year (applied for patients with septic shock)	17.5% (Karlsson et al^18^)	
Readmission first year (all sepsis patients)	20.6% (Zilberberg et al^20^)	
ICU days	Patients with sepsis Septic shock, 7.4 days (Ferrer et al^10^) No septic shock, 2.0 days (Ferrer et al^10^) Patients without sepsis True negatives, 1.0 days (Swedish registry data^21^) False positives, 2.0 days (assume same as “no septic shock”)	
Ward days	Patients with sepsis Septic shock, 17.0 days (Ferrer et al^10^) No septic shock, 5.7 days (Ferrer et al^10^) Patients without sepsis True negatives, 5.7 days (assume same as “no septic shock”) False positives, 5.7 days (assume same as “no septic shock”)	
Long-term consequences for patients with sepsis (frequency)	Impaired kidney function (14.1%) Amputation (8.5%) Depression (2.8%) PTSD (9.9%) (York Health Economics Consortium^22^)	
Long-term survival	Patients with sepsis RR vs general population (Linder et al^23^) Patients without sepsis General population survival	
Discount rate	3.0%	

Abbreviations: ICU, intensive care unit; PTSD, post-traumatic stress disorder; RR, relative risk; SOC, standard of care.

The sensitivity and specificity of the respective screening tool divided patients further as either “true positives” or “false negatives” for patients that develop sepsis, or “true negatives” or “false positives” for patients that do not develop sepsis during their ICU stay (Figure 1). In Sweden, sepsis screening tools are not routinely used in clinical practice, and therefore the Swedish model base case evaluates the sepsis prediction algorithm in comparison with sepsis diagnosis. Other comparators are tested in scenario analyses (eg, the illness severity risk scores [SOFA]^11^ and the National Early Warning Score [NEWS2]).^24^ NEWS2 has been endorsed for use by NHS England and NHS Improvement in acute and ambulance settings to identify acute deterioration, including sepsis.^25^

The potential of an algorithm to improve early detection of sepsis was integrated in the model through the risk of septic shock and in-hospital mortality. To model these time-dependent variables, the sum of 2 components (mean hours to sepsis detection and mean hours to empiric antibiotic from detection) was used to estimate the total hours to treatment. In the model base case, we assumed that the mean time to empiric antibiotic from diagnosis is 0 hours (ie, ICU patients receive treatment immediately when sepsis is diagnosed by healthcare professionals). This is not necessarily in line with the timing of empiric antibiotic administration seen in clinical practice but will not affect the comparison since the same assumption is made for both comparison arms. As mentioned above, the NAVOY® Sepsis algorithm has been validated in a prospective randomized clinical trial, showing that the algorithm detects sepsis up to 3 hours earlier than onset of sepsis in accordance with Sepsis-3 criteria.^11^ In practice, patients are not diagnosed at the same time as the Sepsis-3 criteria are fulfilled; it can take hours before the condition is discovered and diagnosed. The use of a sepsis prediction algorithm does not imply that the patient will be diagnosed with sepsis on the sole basis of an alert from the algorithm. However, it is reasonable to assume that if a suspicion of sepsis can be made 3 hours sooner than it is currently, the whole process can be accelerated in at least the same amount of time (ie, -3 total hours to treatment compared with diagnosis for current practice). Hence, in the model base case, true positives screened with the sepsis prediction algorithm were assumed to receive treatment 3 hours earlier than “true positives” for current practice. For the “false negatives,” the appropriate treatment was assumed to be delayed by 3 extra hours (ie, +3 hours to treatment compared with diagnosis for current practice). Since there is uncertainty with regard to the time to correct diagnosis in clinical practice, scenario analyses were conducted where the time to diagnosis is varied in the -4 hour to -1 hour interval.

The risk of developing septic shock was assumed to be a continuous variable determined by the total hours to treatment compared with diagnosis for current practice. In the model base case, the risk of septic shock in current practice was estimated to be 56.6% based on the findings by Sakr et al.^2^ It is arbitrarily assumed that the risk of septic shock for a 3-hour earlier detection is around 40% and that the risk of septic shock per extra hour of delayed treatment thereafter increases linearly by approximately 5.5 percentage points per hour ([0.566-0.4]/3 = 0.055). Scenario analyses were performed with other risks for septic shock, as well as testing for a constant risk for septic shock, independent of time to treatment.

The risk for in-hospital mortality was also a continuous variable determined by the total number of hours to treatment compared with diagnosis for current practice. The time-dependent in-hospital mortality was extrapolated from the results of Ferrer et al,^10^ and different risk equations were estimated for patients who develop or who do not develop septic shock. The risk equations were estimated in 2 steps. In step 1, a linear programming function was set with restrictions to solve for the percentage of patients who have died at different time points (hours) after diagnosis. In step 2, we used the point estimates from step 1 to fit parametric curves that describe the relationship between mortality (*y*) and hours from diagnosis to antibiotic treatment (*t*) for patients with septic shock (*y* = 0.4281e^0.0272t^) and without septic shock (*y* = 0.0052*t*). This means that the sepsis-related mortality within the first hour after diagnosis is 42.8% and 0.5% for patients with septic shock and without septic shock, respectively. A 3-hour earlier detection, which was used in the base case for the algorithm, would mean that the in-hospital mortality rate for patients with septic shock is 39.5% (0.4281e^0.0272(-3)^). In scenario analyses, alternative methods to estimate in-hospital mortality are tested (eg, a 7.6% linear decrease in survival for septic shock per extra hour of delayed treatment, as reported by Kumar et al,^5^ and a constant mortality risk of 33% for patients with septic shock and 22% for sepsis patients with no septic shock, independent of time to treatment, as reported by Lengquist et al^19^). For patients without sepsis, no assumptions of in-hospital survival were made due to the diversity of other diagnoses in ICUs. This will not impact the results since the proportion of patients without sepsis is the same for both comparison arms.

Due to a lack of data that distinguish patients with and without septic shock with regard to readmissions during the first year, we conservatively assumed the same readmission rate for the 2 categories of patients. Zilberberg et al^20^ estimate the readmission rate independent of sepsis severity to be 20.6%, which is used here. To consider mortality the first year after discharge for patients with septic shock, we used the estimate from Karlsson et al,^18^ who estimate the first-year post-discharge mortality at 17.5%.

In years subsequent to the first year, long-term survival and long-term consequences were considered. Patients without sepsis were assumed to have the same long-term survival as the general population (which does not impact the incremental survival since it is the same for both arms), whereas relative risks on the general population’s long-term survival were applied for patients who enter the model with sepsis. The relative risks of long-term survival applied in the analysis are 5.5 in years 1 to 5, 3.1 in years 6 to 10, and 1.0 from year 11 onward, in accordance with Linder et al.^23^ Scenario analyses are conducted where the long-term survival for patients with sepsis is assumed to be the same as for the general population. Long-term consequences were included for patients with sepsis and applied after the first year. Long-term consequences (with associated frequencies) included impaired kidney function (14.1% of patients), amputation (8.5%), depression (2.8%), and post-traumatic stress disorder (9.9%).^22^

The number of ICU and hospital ward days were estimated based on the findings of Ferrer et al.^10^ We used their presented medians and standard deviations to provide γ distributed simulations of ICU and hospital ward days. We assumed that the shortest length of ICU stay belongs to sepsis patients without septic shock, reflecting the proportion of patients without septic shock presented in Ferrer et al^10^ and vice versa for patients with septic shock. We thereafter calculated the mean number of ICU and hospital days for each group separately. For sepsis patients with septic shock, the number of ICU and hospital ward days were estimated to be 7.4 days and 17.0 days, respectively. The corresponding numbers for patients with sepsis but without septic shock were estimated to be 2.0 days and 5.7 days, respectively. For patients without sepsis, the number of ICU days for true negatives was estimated to be 1.0 days in accordance with Swedish Intensive Care Registry data^21^ and 2.0 days for “false positives,” reflecting the number of ICU days for sepsis patients without septic shock.^10^ The number of hospital ward days for these groups of patients were assumed to be the same as the number of days for sepsis patients without septic shock (ie, 5.7 days). The total cost for ICU and hospital ward for each comparator arm was calculated by multiplying the number of days with a unit cost for each ward type (**Supplementary Table S1**).^26^ The cost for the sepsis prediction algorithm was based on the cost per patient divided by the incidence of sepsis resulting in the cost to detect 1 sepsis patient with the technology.

**Figure 2.**
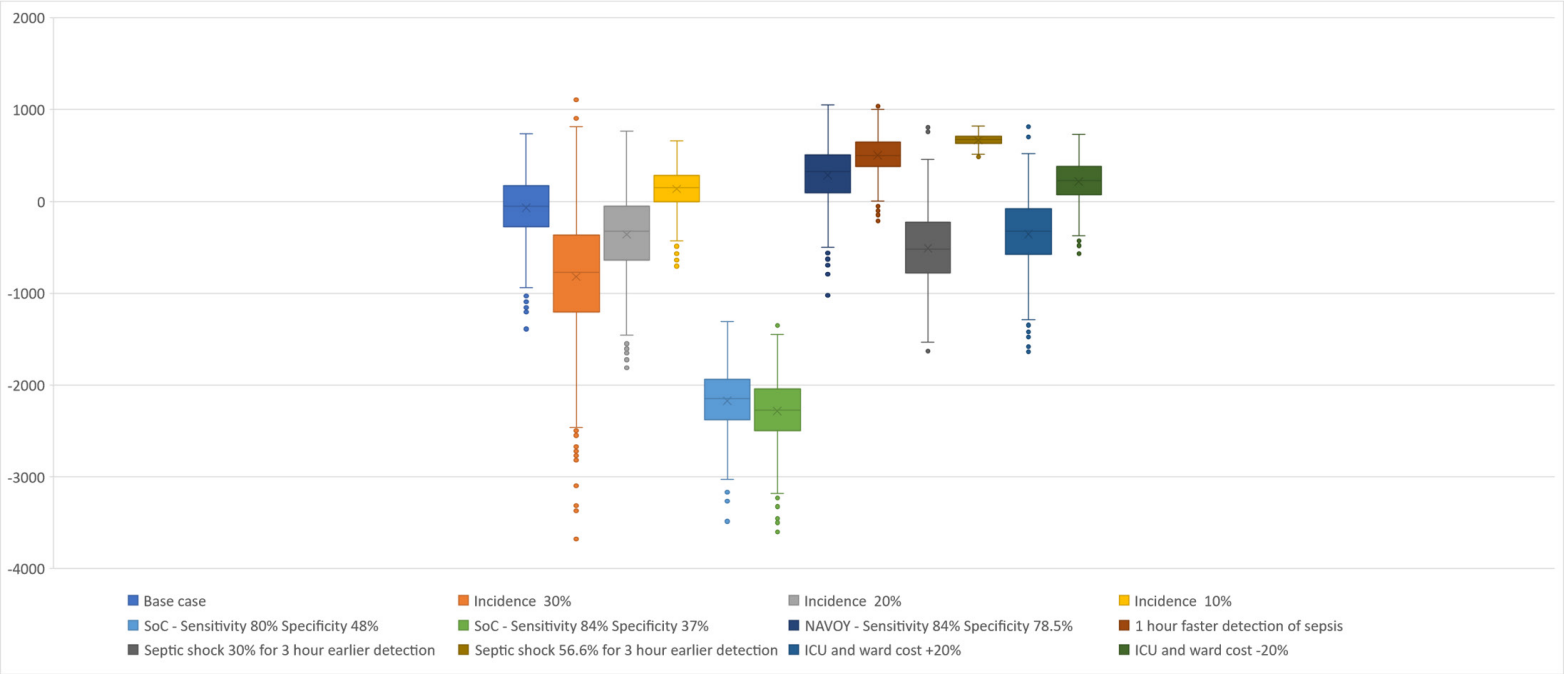
Stochastic Analysis of Cost Impact and Cost-effectiveness Abbreviations: ICU, intensive care unit; SoC, standard of care. Values >0 reflect cost increases per patient; values <0 reflect cost savings per patient.

Calculations were conducted on a per-patient level (average cost per patient) and on a country level (total potential cost impact for Sweden). Both deterministic and stochastic analyses were conducted. The stochastic analyses were based on 1000 simulations using information about each variable’s mean, standard error, and statistical distribution (eg, γ, β, log-normal); details are provided in Table 2 and Figure 2.^27^ The cost calculations were conducted using the euro as the common currency, as it facilitates cross-country comparisons (1 Sk = €0.098 using the exchange rate as of December 14, 2020). We used the Consolidated Health Economic Evaluation Reporting Standards (CHEERS) checklist when writing our report.^28^

**Table 2.** Parameters Used in the Stochastic Sensitivity Analysis

**Item**	**Mean**	**SE**	**Distribution**	**Source**
Incidence (%)	14.10	0.0038	β	Derived from Sakr et al^2^
Sensitivity and specificity (%)				
Sensitivity NAVOY® Sepsis	80.00	0.0183	β	Approximated based on Lenquist et al^19^
Specificity NAVOY® Sepsis	85.10	0.0068	β	Approximated based on Lenquist et al^19^
Sensitivity sepsis diagnosis in clinical practice	79.20	0.0181	β	Derived from Lenquist et al^19^
Specificity sepsis diagnosis in clinical practice	78.50	0.0063	β	Derived from Lenquist et al^19^
Hours to treatment				
Mean hours to sepsis diagnosis	Range, -3 to 3	0.5	Normal	Assume 0.5 hours from mean value
Mean hours to empiric antibiotic	0	0.5	Normal	Assume 0.5 hours from mean value
Septic shock				
Septic shock when diagnosis set 3 hours earlier	40.00%	0.04	β	Assume 10% of mean value
Proportion septic shock, current SOC	56.60%	0.0091	β	Derived from Sakr et al^2^
In-hospital mortality				
Septic shock: RR coefficient vs extrapolated value	1.0	0.255	Log-normal	Derived to match mortality in Sakr et al^2^
No septic shock: RR coefficient vs extrapolated value	1.0	0.588	Log-normal	Derived to match mortality in Sakr et al^2^
Long-term mortality				
HR year 1-5, <60	17.8	2.653	Log-normal	Derived from Linder et al^23^
HR year 5-10, <60	6.0	1.301	Log-normal	Derived from Linder et al^23^
HR year 1-5, 60-70	5.5	1.250	Log-normal	Derived from Linder et al^23^
HR year 5-10, 60-70	3.1	0.791	Log-normal	Derived from Linder et al^23^
HR year 1-5 ,>70	2.4	0.791	Log-normal	Derived from Linder et al^23^
HR year 1-5 ,>70	1.8	1.684	Log-normal	Derived from Linder et al^23^
ICU days				
Sepsis: Septic shock	7.4	0.1989	γ	Derived from Sakr et al^2^
Sepsis: No septic shock	2.0	0.1650	γ	Derived from Sakr et al^2^
No sepsis: True negatives	1.0	0.0264	γ	Derived from Sakr et al^2^
No sepsis: False positives	2.0	0.0264	γ	Assume same as "No sepsis: True negatives"
Ward days				
Sepsis: Septic shock	17	0.4570	γ	Assume relative SE based on ICU days
Sepsis: No septic shock	5.7	0.4703	γ	Assume relative SE based on ICU days
No sepsis: True negatives	5.7	0.1505	γ	Assume relative SE based on ICU days
No sepsis: False positives	5.7	0.1505	γ	Assume same as "No sepsis: True negatives"
Treatment outcomes				
Postdischarge mortality for patients with septic shock	17.50%	0.0175	β	Derived from Karlsson et al^18^
Readmission (first year)	20.60%	0.0042	β	Derived from Zilberberg et al^20^
Utility				
Utility decrement year 1-5	-0.164	0.0395	Normal	Derived from Cuthbertson et al^27^
Utility decrement year 6-10	-0.124	0.0394	Normal	Derived from Cuthbertson et al^27^
Utility decrement year 11+	0	0	Normal	Assume same utility as general population after 11+ years
Long-term consequences				
Impaired kidney function	14.10%	0.0044	β	Derived from York Health Economics Consortium^22^
Amputation	8.50%	0.0035	β	Derived from York Health Economics Consortium^22^
Depression	2.80%	0.0021	β	Derived from York Health Economics Consortium^22^
PTSD	9.90%	0.0038	β	Derived from York Health Economics Consortium^22^

Abbreviations: HR, hazard ratio; ICU, intensive care unit; PTSD, post-traumatic stress disorder; RR, relative risk; SE, standard error; SOC, standard of care.

## RESULTS

### Deterministic Base Case Analysis

Using the methodology described above, we quantified the potential financial and medical advantages using a predictive algorithm such as NAVOY® Sepsis over the current clinical practice for sepsis diagnosis in Sweden. The results are illustrated in Table 3.^29,30^ From the model, we calculated average total costs for both diagnostic procedures and incremental costs (cost savings) for an ICU patient. The aggregated cost savings were derived by multiplying the average results with the yearly number of patients admitted to ICUs. In Sweden, the yearly attendance to ICUs is estimated to be 45 000 patients.^21^ Of these, approximately 36 900 patients do not have sepsis upon admission.^2^ Setting the cost per patient per ICU day with the sepsis prediction algorithm to €90, for example, would render a per-patient cost of €146, considering an average of 1.62 days in the ICU and €1037 per sepsis patient detected (average days in the ICU divided by the sepsis incidence).

**Table 3.** Model Base Case Results

	**Per ICU Patient**			**National Aggregated Results**
	**Sepsis Prediction Algorithm**	**Current Practice**	**Incremental**	**Potential Cost Impact (€)**
Prediction costs (per sepsis patient)		—		
Sepsis prediction algorithm (€)	1037		1037	38 268 415
Direct costs (€)				
Hospitalization (ward)	4035	4169	-134	-4 954 159
Hospitalization (ICU)	10 322	11 331	-1009	-37 227 454
Readmission	835	826	8	305 598
Long-term consequences (€)	208	186	22	808 685
Total costs (€)	16 436		-76	-2 798 915
**Outcome**				**Outcome Gains**
Length of stay (ward), in days	6.45	6.66	-0.21	7916 ward days
Length of stay (ICU), in days	1.62	1.78	-0.16	5860 ICU days
Readmissions (%)	20.0	19.8	0.2	—
In-hospital mortality (%)	2.8	3.7	-1.0	356 lives

Abbreviation: ICU, intensive care unit.

Under these assumptions, a machine learning algorithm that can detect sepsis 3 hours before current practice will reduce the cost per ICU patient by 0.5%. The total cost per patient with such an algorithm is €16 436, and the cost per patient for current practice is €16 512. The potential cost savings per patient is thus €76, and the aggregated yearly cost saving for the Swedish healthcare system is €2 798 915.

The largest cost savings are due to a shorter average length of stay in the ICU (0.16 days shorter for an algorithm like NAVOY® Sepsis compared with current practice), resulting in a cost saving of 8.9% (€10 322 vs €11 331) per patient related to ICU hospitalization. The shorter length of stay in ICU for the sepsis prediction algorithm compared with current practice results in 5860 fewer ICU days per year on an aggregated national level. In addition to the reductions in resources used, faster detection also implies reduced in-hospital mortality, resulting in 356 lives saved per year in Sweden alone.

### Deterministic Scenario Analyses

Scenario analyses were run for the categories of model parameters presented in Table 4. The most sensitive parameters are the incidence, sensitivity, and specificity inputs and the probability of septic shock with the prediction algorithm. Aggregated cost savings would be about 11 times greater (1033%) if the ICU sepsis incidence is 30% (compared with 14.1% in the model base case). Assuming a sensitivity and specificity for the comparator based on NEWS2, as presented by Mellhammar et al^30^ (sensitivity 0.84, specificity 0.37), there is an aggregated yearly cost saving of €84 956 039, which constitutes approximately a 30-fold increase (2935%) compared with the base case (current practice). If the proportion of septic shock is set to 30% (40% in model base case) when sepsis detection is set 3 hours earlier, the cost savings would be 7 times greater (603%). Assuming that the proportion of septic shock is time independent and the same as for current practice, no cost savings would be achieved. The model is also sensitive to the time to sepsis detection. If the time to sepsis detection for the prediction algorithm is set 1 hour earlier (4 hours instead of 3 hours), the cost savings would be 4 to 5 times greater (329%). If the time to sepsis detection for the prediction algorithm is less than 2 hours earlier, no cost savings would be achieved.

**Table 4.** Scenario Analyses

**Scenario**	**Potential Cost Impact (€), as Cost Savings in Relation to Base Case^a^)**
Base case	-2 798 915
Incidence (BC, 14.1%)	
Incidence 30%	-31 707 067 (+1033%)
Incidence 20%	-13 525 839 (+383%)
Incidence 10%	4 655 389 (-266%)
Sensitivity and specificity for comparator (BC, 79.2% and 78.5%, sepsis diagnosis in clinical practice)	
Sensitivity and specificity for SOFA (80.0% and 48.0%); Desautels et al^29^	-64 124 165 (+2191%)
Sensitivity and specificity for NEWS2 (84.0% and 37.0%); Mellhammar et al^30^	-84 956 039 (+2935%)
Sensitivity and specificity for NAVOY® Sepsis (BC, 80.0% and 85.1%)	
Sensitivity (BC) and specificity (Sepsis-3) (80.0% and 78.5%)	10 457 010 (-474%)
Time to sepsis detection of prediction algorithm (BC, -3 hours)	
-4 total hours to treatment compared with time to diagnosis for current practice	-12 000 432 (+329%)
-2 total hours to treatment compared with time to diagnosis for current practice	6 389 236 (-328%)
-1 total hours to treatment compared with time to diagnosis for current practice	15 563 483 (-656%)
Probability of septic shock with prediction algorithm (BC, 40%)	
Proportion septic shock is 30%	-19 671 814 (+603%)
Proportion septic shock is 56.6%	25 210 099 (-1001%)
In-hospital mortality (BC time-dependent extrapolated from Ferrer et al^10^	
In-hospital mortality: Decrease in survival for septic shock 7.6% per hour (set to 40% at 0 hours); Kumar et al^5^	-2 035 611 (-27%)
In-hospital mortality for septic shock 33% and 22% for no septic shock; Lengquist et al^19^	-3 538 803 (+26%)
Length of stay in ICU and hospital ward (BC)^b^	
Length of stay ICU and ward day increase by 25%	-13 344 318 (+377%)
Length of stay ICU and ward day decrease by 25%	7 746 489 (-377%)
Postdischarge first year	
Septic shock postdischarge mortality first year 0% (BC, 17.5%)	-2 915 397 (+4%)
Readmission rate 0% (BC, 20.6%)	-3 104 513 (+11%)
Readmission rate 40% (BC, 20.6%)	-2 511 118 (-10%)
Unit costs for hospital (BC)^c^	
Unit cost of ICU and ward day increase by 25%	-13 344 318 (+377%)
Unit cost of ICU and ward day decrease by 25%	7 746 489 (-377%)
Unit cost of readmission increase by 25% (BC, €4166)	-2 722 515 (-3%)
Unit cost of readmission decrease by 25% (BC, €4166)	-2 875 314 (+3%)
Long-term effects	
Long-term survival for patients with sepsis same as general population (BC, see Linder et al^23^)	-2 139 804 (-24%)
Long-term consequences not included (BC, included)	-3 607 600 (+29%)
Model time horizon 1 year (BC, lifetime)	-3 607 600 (+29%)
Discount rate 0% (BC, 3%)	-2 682 947 (-4%)

Abbreviations: BC, base case; ICU, intensive care unit.

^a^The value in parentheses shows the percentage change in comparison with the base case. A plus sign (+) denotes increased cost savings; a minus sign (-) denotes decreased cost savings (or increased costs).

^b^ICU: Septic shock, 7.4 days; no septic shock, 2.0 days; true negatives, 1.0 days; false positives, 2.0 days. Ward days: Septic shock, 17.0 days; no septic shock, 5.7 days; true negatives, 5.7 days; false positives, 5.7 days.

^c^ICU, €6353; hospital ward, €626.

Considering that sepsis detection in clinical practice does not concur with the time as the Sepsis-3 criteria are fulfilled, there is most likely a gap between the earliest time when the diagnosis is possible to establish (in accordance with the criteria) and the time when the diagnosis is established in practice, and this gap will add to the total time to treatment and thus increase the cost savings. In other words, the potential effect the prediction algorithm could have on time to treatment is most likely underestimated. The sensitivity analyses also show the results to be sensitive to changes in the cost and length of stay in ICU and hospital ward. Increasing the length of stay and cost in ICU and hospital ward by 25% would increase the cost savings by 377%. Decreasing the length of stay and cost in ICU and hospital ward by 25% would result in no cost savings.

### Stochastic Sensitivity Analysis

To investigate how the cost estimates for the different scenarios were distributed, we conducted stochastic analyses based on 1000 individual simulations for the 11 most influential parameters, including the base case (**Supplementary Table S2**). When conducting the stochastic analysis, we kept each individual parameter of interest constant at its mean and allowed all other parameters to vary stochastically. By doing this, we were able to investigate the impact the parameter had on the overall uncertainty (vs the base case). We also estimated the incremental cost-effectiveness ratios (ICER = [Total Cost (NAVOY® Sepsis) – Total Cost (clinical practice)]/[QALYs (NAVOY® Sepsis) – QALYs (standard of care)]) and evaluated the percentage of the simulations below a cost per quality-adjusted life-year (QALY) threshold of €20 000 and €50 000, respectively. The results are detailed in Figure 2 as box plots and as descriptive statistics.

Figure 2 shows that there is cost saving per patient in all scenarios except when (1) sepsis accounts for less than 10% of all ICU admissions without sepsis at admission, (2) detection of sepsis is only 1 hour faster with NAVOY® Sepsis vs current clinical practice, (3) sensitivity is increased to 84% (NEWS2) and specificity is reduced to 78.5% (SOFA), (4) the prevalence of septic shock is the same (56.6%) despite 3 hours earlier detection, and (5) the unit costs for ICU and hospital ward is reduced by 25%. There are 2 scenarios in which NAVOY® Sepsis is unattractive from a cost-effectiveness perspective (>€20 000/QALY): when detection is only 1 hour faster and when it is assumed that faster detection does not impact the prevalence of septic shock.

The consequence of a test’s specificity is clearly illustrated in Figure 2; when the specificity of the comparator in clinical practice is reduced from 78.5% (Sepsis-3 criteria) to 48% (SOFA) and to 37% (NEWS2), the cost saving increased from €76 to €2170 and €2283, respectively. Likewise, if the specificity of NAVOY® Sepsis is reduced from 85.1% to 78.5% (Sepsis-3), the cost saving per patient of €76 will instead result in a cost increase of €287 per patient.

## DISCUSSION

In this study, the potential cost savings and cost-effectiveness achieved by a machine learning algorithm forecasting the onset of sepsis were calculated using a novel health economic model developed for this purpose. The model shows that cost savings can be achieved for the healthcare sector in terms of factors such as reduced in-hospital mortality and shorter ICU length of stay. The model base case, with conservative assumptions about sepsis incidence and time to treatment, shows that by using a sepsis prediction algorithm that can detect sepsis 3 hours earlier than current practice, the average ICU length of stay can be reduced by 0.16 days. This might sound like a small amount, but it aggregates to a total of 5860 fewer ICU days per year on a national level. In addition to reducing costs, this would reduce the ICU occupancy rate and perhaps relieve some of the stress for the ICU staff. The base case model result also shows a potential of saving 356 lives per year in Sweden, almost 1 life per day. This can be compared to approximately 200 people in Sweden being killed in traffic each year.^31^ The Swedish Transport Agency has estimated the value of a “statistical life” to approximately €4 million,^32^ which is equivalent to a value of a quality-adjusted life-year (QALY) of €235 200.^33^

### Strengths and Limitations

The results are sensitive to the sepsis incidence, which, as discussed by Bray et al,^16^ depends, among other things, on how sepsis is diagnosed in the clinic. A broad definition of sepsis leads to a higher incidence, and a prediction algorithm would thus have a larger potential of reducing costs in situations where a broader definition is used. Internationally, the Sepsis-3 criteria^11^ are acknowledged as the gold standard when diagnosing sepsis, but these criteria are not widely used in practice. Lengquist et al^19^ showed that sepsis is underdiagnosed in Swedish ICUs, indicating that a sepsis prediction algorithm has a high potential of contributing to identifying patients with sepsis. Further, patients admitted with sepsis are not included in the model even though they can develop a secondary sepsis infection during the ICU stay.^17^ This means that a sepsis prediction algorithm would have a potential of reducing costs also for this group of patients, adding to the total cost savings presented in this study. The results are also sensitive to the time to diagnosis, which in turn affects time to treatment. Since sepsis is underdiagnosed in practice,^19^ it is reasonable to assume that a sepsis prediction algorithm has the potential to substantially shorten the time to treatment compared with current practice. Also, the algorithm was developed on a large database of critically ill patients. The model base case was set to be conservative in relation to both incidence and time to diagnosis, to avoid exaggerating the positive effects of the use of a sepsis prediction algorithm like NAVOY® Sepsis.

Whereas previous research has primarily focused on interventions for sepsis treatment,^34^ our study is the first that has investigated the potential cost impact and cost-effectiveness of implementing a machine learning algorithm for earlier detection of sepsis. Most of the studies reviewed by Higgins et al^34^ were cost-effectiveness studies with a positive incremental cost-effectiveness ratio (extra benefits at extra costs), indicating that interventions for sepsis rarely are cost saving. In the present study, we also assessed the probabilistic cost-effectiveness of the machine learning algorithm using various alternative assumptions regarding sepsis incidence rates, rates of sensitivity and specificity, hours of faster sepsis detection, prevalence of septic shock, and unit costs of an ICU stay. Generally, we found that NAVOY® Sepsis was a dominant alternative (cost saving and more effective) compared with current clinical practice. We found that NAVOY® Sepsis was not cost-effective (threshold >€20 000) in 2 of the 11 tested scenarios; when the detection was only 1 hour faster instead of 3 hours, and when the prevalence of septic shock at 3-hour faster detection was 56.6% instead of 40%. Since the former assumption must be regarded as highly conservative and the latter is unrealistic, as it implies no gains from earlier detection, we regard these results as indicators that NAVOY® Sepsis has a high probability of being a cost-effective technology.

This study shows that future clinical studies of prediction algorithms such as NAVOY® Sepsis need to be powered to provide stable results of specificity. The reason is that “false alarms” have an impact on ICU days and thereby unnecessary ICU resources for false-positive patients. Running model simulations of a 78.5% specificity instead of 85.1% reduced the cost offsets per patient for ICU from €650 to €1009. Thus, a more specific instrument is more important than a more sensitive one as it has a greater impact on resource consumption. The base case for the model is based on international data sources (eg, Ferrer et al^10^ and Sakr et al^2^), which might not be directly applicable to a Swedish setting. This fact is accounted for in the sensitivity analyses, where alternative scenarios for parameters that could differ between countries (eg, incidence, length of stay, in-hospital mortality, unit costs) were accounted for. Our analysis indicates that different countries’ definitions of sepsis could have a substantial impact on incidence and thereby the cost-saving potential of introducing an algorithm, whereas differences in unit costs will have a minor impact. Also, the results of this study apply to Sweden, a relatively small, technologically advanced country. The benefits of a sepsis prediction algorithm would naturally apply to countries other than Sweden but on a larger or smaller scale, depending on the population size. The implementation of a prediction algorithm, however, would require systems for electronic data capture, which is not yet available in all countries.

## CONCLUSION

Our health economic model shows that a machine learning prediction algorithm that can find septic patients 3 hours earlier than what is possible today can save lives, reduce time spent in the ICU, and reduce costs for society and the healthcare sector. The value of saving over 350 lives adds up to a total of €1.4 billion, based on the Swedish Transport Agency’s estimate of a statistical life.^32^ Internationally, the benefits of a sepsis prediction algorithm would be similar to those for Sweden but on a larger or smaller scale, depending on population size.

The sensitivity analyses show that the prevalence of septic shock has a large impact on the results, and there is little found in the literature on the possibility of reducing the septic shock rate by early detection of sepsis. Further research on the relationship between the key events in the management of sepsis, such as time of sepsis detection in clinical practice, time of fulfillment of Sepsis-3 criteria, time to treatment, and development of septic shock, would be valuable to develop an even more granular model. Future research should also focus on patient categories with high levels of inflammation, such as burns, trauma, and pancreatitis, where sepsis is known to be very difficult to diagnose and where a prediction algorithm could be of immense value.

### Disclosures

The Swedish Institute for Health Economics (IHE), where OE and JH are employed, has received compensation from AlgoDx AB to conduct the research upon which this manuscript is based. AlgoDx AB is also sponsoring an ongoing study for prospective evaluation of the algorithm used in this manuscript, and FS’s institution is reimbursed for costs related to performing that study. IP and JS are shareholders of AlgoDx AB.

### Author Contributions

OE designed the model together with JH, performed the computations, drafted the manuscript, discussed the results, and contributed to the final manuscript. JH designed the model together with OE, drafted the manuscript, discussed the results, and contributed to the final manuscript. FS was involved in discussions and interpretations, drafted parts of the manuscript, discussed the results, and contributed to the final manuscript. IP was involved in discussions and interpretations, supervised the work, drafted the manuscript, discussed the results, and contributed to the final manuscript. JS conceived the idea, participated in planning and design of the study, supervised the work, discussed the results, and contributed to the final manuscript.

## Supplementary Material

Online Supplementary Material
