# Supplementary material for: The Potential Cost and Cost-Effectiveness Impact of Using a Machine Learning Algorithm for Early Detection of Sepsis in Intensive Care Units in Sweden
Source: J Health Econ Outcomes Res. 2022 Apr 26;9(1):101–10. doi: 10.36469/jheor.2022.33951 (PMC9042649; doi:10.36469/jheor.2022.33951)
Supplement: Online Supplementary Material [file jheor_2022_9_1_33951_88834.pdf]

Journal of Health Economics and Outcomes Research

Online Supplementary Material

The potential cost and cost-effectiveness impact of using a machine learning algorithm for early detection of sepsis in intensive care units in Sweden. *JHEOR*. **2022;9(1):101-110.** doi:10.36469/jheor.2022.33951

Table S1: Unit Costs of Healthcare Resource Utilization

Table S2: Stochastic Analysis of Cost Impact and Cost-effectiveness

This supplementary material has been provided by the authors to give readers additional information about their work.

This is an open-access article distributed under the terms of the Creative Commons Attribution 4.0 International License (CCBY-4.0). View this license's legal deed at http://creativecommons.org/licenses/by/4.0 and legal code at http://creativecommons.org/licenses/by/4.0/legalcode for more information.

Table S1. Unit Costs of Healthcare Resource Utilization

	Cost (€)	Source
Hospital unit costs		
ICU cost/day	6353[a]	The Swedish Intensive Care Registry[25]; cost per day: 50 000 Sk-80 000 Sk ~65000 Sk
Ward cost/day	626[a]	Swedish Southern Healthcare Region price list[26]; VD010 Nursing day cost infectious diseases: 6403 Sk
Readmission	4166[a]	Swedish Southern Healthcare Region price list[26]; S59E Other infectious and parasitic diseases U: 42 618 Sk
Diagnostics (price per ICU bed per day)		
Sepsis prediction algorithm	90	Assumption
Long-term consequences (annual cost)		
Impaired kidney function	938[b]	York Health Economics Consortium[24]; annual cost for CKD or renal failure: £801 per year
Amputation	898[b]	York Health Economics Consortium[24]; NICE type 2 diabetes guidelines estimates a yearly cost of £767
Depression	563[b]	York Health Economics Consortium[24]; NICE guideline cost of antidepressant treatment: £481
PTSD	1296[b]	York Health Economics Consortium[24]; NICE guideline cost of PTSD: £1107

Abbreviations: CKD, chronic kidney disease; NICE, National Institute for Health and Care Excellence; PTSD, post-traumatic stress disorder.

[a] Exchange rate €/Sk=0.098 (as of December 14, 2020).

[b] Exchange rate €/£=1.10 (as of December 14, 2020) and inflated to 2019 prices.

Table S2. Stochastic Analysis of Cost Impact and Cost-effectiveness

	**Base Case**	**Incidence 30%**	**Incidence 20%**	**Incidence 10%**	**SOC Sens, 80%; Spec, 48%**	**SOC Sens, 84%; Spec, 37%**	**NAVOY® Sepsis Sens, 84%; Spec, 78.5%**	**1 h Faster Detection of Sepsis**	**Septic Shock 30% for 3 h Earlier Detection**	**Septic Shock 56.6% for 3 h Earlier Detection**	**ICU and Ward Cost +25%**	**ICU and Ward Cost -25%**
Mean (€)	-68	-820	-358	136	-2170	-2283	287	503	-509	668	-355	217
Median (€)	-48	-773	-322	153	-2147	-2273	325	501	-518	673	-325	230
Low 95% CI (€)	-773	-2319	-1267	-320	-2900	-2966	-378	68	-1276	544	-1197	-260
High 95% CI (€)	504	270	398	521	-1581	-1729	798	827	243	773	291	581
IQR (Q3-Q1) (€)	450	836	587	290	437	456	409	261	550	78	493	306
Percent cost <€0 (cost saving)	55.3	91.1	79.2	25.3	100.0	100.0	16.7	0.9	90.9	0.0	82.7	16.2
ICER, per QALY (€)	-1802	-9595	-6139	4226	-52 309	-59 384	6883	46608	-8407	101948	-8591	4987
Percent <€20,000 threshold	100	100	100	86	100	100	98	21	99	0	98	87
Percent <€50,000 threshold	100	100	100	97	100	100	100	52	100	7	100	99

Abbreviations: CI, confidence interval; ICER, incrmental cost-effectiveness ratio; ICU, intensive care unit; IQR, interquartile range; Q, quarter; QALY, quality-adjusted life-year; Sens, sensitivity; SOC, standard of care; Spec, specificity.
